# Molecular and Neuroimaging Profile Associated with the Recurrence of Different Types of Strokes: Contribution from Real-World Data

**DOI:** 10.3390/jcm14051460

**Published:** 2025-02-21

**Authors:** Crhistian-Mario Oblitas, Ana Sampedro-Viana, Sabela Fernández-Rodicio, Manuel Rodríguez-Yáñez, Iria López-Dequidt, Arturo Gonzalez-Quintela, Antonio J. Mosqueira, Jacobo Porto-Álvarez, Javier Martínez Fernández, Inmaculada González-Simón, Marcos Bazarra-Barreiros, María Teresa Abengoza-Bello, Sara Ortega-Espina, Alberto Ouro, Francisco Campos, Tomás Sobrino, José Castillo, María Luz Alonso-Alonso, Pablo Hervella, Ramón Iglesias-Rey

**Affiliations:** 1 Neuroimaging and Biotechnology Laboratory (NOBEL), Clinical Neurosciences Research Laboratory (LINC), Health Research Institute of Santiago de Compostela (IDIS), 15706 Santiago de Compostela, Spain; ana.sampedro@rai.usc.es (A.S.-V.); sfernandezrodicio@gmail.com (S.F.-R.); inmaculada.gonzalez.simon@sergas.es (I.G.-S.); marcos.bazarra.barreiros@sergas.es (M.B.-B.); maria.teresa.abengoza.bello@sergas.es (M.T.A.-B.); sara.ortega@rai.usc.es (S.O.-E.); jose.castillo.sanchez@sergas.es (J.C.); maria.luz.alonso.alonso@sergas.es (M.L.A.-A.); pablo.hervella.lorenzo@sergas.es (P.H.); 2 Stroke Unit, Department of Neurology, Hospital Clínico Universitario, 15706 Santiago de Compostela, Spain; manuel.rodriguez.yanez@sergas.es; 3 Department of Neurology, Hospital Clínico Universitario de Ferrol, 15405 Ferrol, Spain; iriaalejandralopez@googlemail.com; 4 Department of Internal Medicine, Hospital Clínico Universitario, 15706 Santiago de Compostela, Spain; arturo.gonzalez.quintela@sergas.es; 5 Department of Radiology, Hospital Clínico Universitario, Health Research Institute of Santiago de Compostela (IDIS), 15706 Santiago de Compostela, Spain; drmosqueiramartinez@gmail.com (A.J.M.); jacoporto@hotmail.com (J.P.-Á.); javier.martinez.fernandez@sergas.es (J.M.F.); 6 Neuroradiology Group, Health Research Institute of Santiago de Compostela (IDIS), 15706 Santiago de Compostela, Spain; 7 NeuroAging Laboratory Group (NEURAL), Clinical Neurosciences Research Laboratory (LINC), Health Research Institute of Santiago de Compostela (IDIS), 15706 Santiago de Compostela, Spain; alberto.ouro.villasante@sergas.es (A.O.); tomas.sobrino.moreiras@sergas.es (T.S.); 8 Centro de Investigación Biomédica en Red de Enfermedades Neurodegenerativas, Instituto de Salud Carlos III, 28029 Madrid, Spain; 9 Translational Stroke Laboratory (TREAT), Clinical Neurosciences Research Laboratory (LINC), Health Research Institute of Santiago de Compostela (IDIS), 15706 Santiago de Compostela, Spain; francisco.campos.perez@sergas.es

**Keywords:** biomarkers, interleukin 6, leukoaraiosis, secondary prevention, stroke recurrences

## Abstract

**Objective:** This study aimed to investigate potential specific molecular and neuroimaging biomarkers for stroke subtype recurrence to improve secondary stroke prevention. **Methods:** A retrospective analysis was conducted on a prospective stroke biobank. The main endpoint was to evaluate the association between different biomarkers and the recurrence of stroke subtypes. Serum levels of interleukin 6 (IL-6) and tumor necrosis factor-alpha (TNF-a) were analyzed as inflammation biomarkers; N-terminal pro-B-type natriuretic peptide (NT-pro-BNP) and microalbuminuria were used as atrial/endothelial dysfunction biomarkers, while leukoaraiosis (LA) and soluble TNF-like inducers of apoptosis (sTWEAK) were used as biomarkers for blood–brain barrier dysfunction. Demographic and clinical variables were also included. **Results:** A total of 5038 stroke patients were included, with a mean follow-up of 4.9 years (±3.3). Stroke recurrences were observed in 18.4% of patients (927 individuals). The main results found were as follows: LA was independently associated with lacunar stroke recurrence (adjusted OR 9.50; 95% CI: 3.12–28.93). NT-pro-BNP levels higher than >1000 pg/mL were independently associated with cardioembolic stroke recurrence (adjusted OR 1.80; 95% CI: 1.23–2.61). Persistently elevated TNF-a levels (>24 pg/mL) after stroke recurrence showed an adjusted OR of 21.26 (95% CI: 12.42–37.59) for atherothrombotic subtype, whereas persistently high sTWEAK levels (>7000 pg/mL) after a second hemorrhagic stroke showed an adjusted OR of 4.81 (95% CI: 2.86–8.07) for hemorrhagic subtype. **Conclusions:** The presence of LA and high levels of NT-pro-BNP, TNF-a, and sTWEAK were associated with an increased risk for lacunar, cardioembolic, atherothrombotic, and hemorrhagic stroke recurrences, respectively.

## 1. Introduction

In the last three decades, there has been a significant decrease in mortality due to advances in treating and managing the acute phase of stroke [1]. However, stroke recurrences in patients with a first ischemic stroke (IS) event are still high, being around 7% at 3 months and 9–15% in the first year with a maximum of 30–50% at 10 years, depending on the series and the etiology of stroke [2,3,4,5,6], despite the efforts of the control target of traditional clinical risk factors associated with stroke recurrence such as arterial hypertension, diabetes mellitus, dyslipidemia, atrial fibrillation (AF), and previous vascular comorbidities, among others [2,7]. Additionally, intracerebral hemorrhage (ICH) showed an overall annual recurrence of 1–3% [8]. Furthermore, recurrent stroke is independently associated with a 2.5- to 3-fold increase in all-cause mortality, along with a significant impact on morbidity [2,9]. Conversely, although fibrinolytic treatment is effective during the acute phase of IS, it does not appear to provide long-term protection against recurrence [10]. Patients who fail to achieve clinical reperfusion also experience poorer outcomes [11].

Early detection and targeted management guided by biomarkers can help personalize prevention strategies and therapeutic interventions, ultimately reducing the risk of recurrence, improving patient outcomes, and alleviating the burden on healthcare systems [1,2,3]. In this context, previous studies have suggested an association between serum and neuroimaging biomarkers and poor short-term outcomes following stroke, including recurrence [12,13,14].

Inflammatory biomarkers such as interleukin-6 (IL-6) and tumor necrosis factor-alpha (TNF-α) have been linked to atherothrombosis [15]. N-terminal pro-B-type natriuretic peptide (NT-pro-BNP) serves as an indicator of a cardiac hemodynamic state, identifying a potential embolic source [16]. The soluble TNF-like weak inducer of apoptosis (sTWEAK) has been associated with blood–brain barrier (BBB) disruption in intracerebral hemorrhage (ICH) and neuroinflammation [17]. Additionally, neuroimaging findings, particularly the presence and severity of leukoaraiosis (LA), have been proposed as markers of chronic BBB dysfunction [14]. However, evidence supporting their role as specific predictors of stroke subtype recurrence remains limited. The main objective of this study was to identify potential molecular and neuroimaging biomarkers linked to long-term stroke subtype recurrence beyond three months post-stroke, aiming to enhance the prediction of specific stroke subtype recurrence.

## 2. Methods

### 2.1. Standard Protocol Approvals, Registrations, and Patient Consent

This study complies with the principles of good clinical practice, which aligns with the Declaration of Helsinki established by the World Medical Association in 1964 and its subsequent update in Fortaleza in 2013. The Galician Ethics Committee approved the biobank “Biobanco Ictus del Complejo Hospitalario Universitario de Santiago” (BICHUS) and the research project (identification code 2019/616). Informed consent was obtained from all participants or their authorized representatives, ensuring the anonymity of all study participants.

### 2.2. Study Design

**Figure 1:**
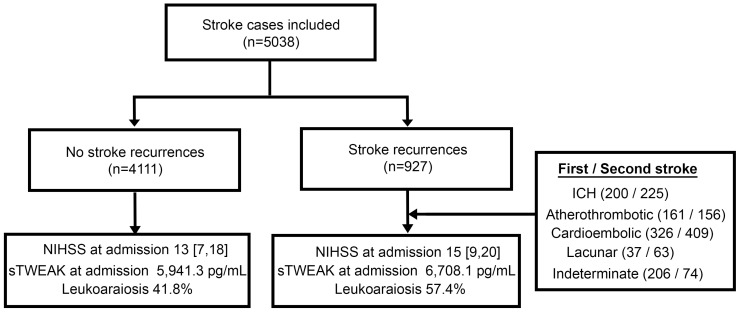
Flowchart for inclusion and exclusion criteria. ICH: intracerebral hemorrhage; NIHSS: National Institutes of Health Stroke Scale; sTWEAK: soluble tumor necrosis factor-like weak inducer of apoptosis.

This study included all patients admitted to the Stroke Unit at Hospital Clínico Universitario de Santiago de Compostela who were prospectively registered in the approved BICHUS biobank. A retrospective analysis was conducted using BICHUS data. The study utilized real-world data, defined as “data relating to patient health status and/or the delivery of health care routinely collected from a variety of sources” [18]. Inclusion criteria comprised patients aged 18 years or older who experienced a first stroke event and were admitted between January 2008 and December 2018. Exclusion criteria included the following: (1) Patients who died during the standardized follow-up period within the first three months after the initial stroke event. (2) Patients without available frozen serum samples for biomarker analysis. These criteria were established to exclude the acute phase of stroke, ensuring a more precise evaluation of factors influencing long-term recurrence. Additionally, including such patients could introduce bias, as their clinical outcomes are shaped by factors distinct from those affecting long-term stroke recurrence [3]. Figure 1 presents a flowchart outlining the study protocol encompassing all stroke subtypes.

Recurrences after the first stroke event were estimated in the same database BICHUS by using electronic medical records searching for re-admission due to a second stroke event in the region’s public network. Patients possibly treated in private centers or outside the region were not registered.

The previous literature has suggested that pro-inflammatory biomarkers (IL-6, TNF-a, and sTWEAK), hemodynamic cardiac hormones (NT-pro-BNP), and neuroimaging biomarkers such as LA might be associated with the risk of stroke recurrences [12,13,14].

### 2.3. Clinical Variables and Neuroimaging Studies

The registry includes demographic variables, vascular risk factors, time from the beginning of the symptomatology until the administration of the reperfusion treatments (fibrinolysis or thrombectomy), comorbidities, associated treatments, axillary body temperature and blood pressure, blood count and coagulation, and biochemical variables. Stroke subtypes included intracerebral hemorrhage (ICH) and IS. The latter was classified in accordance with the TOAST (Trial of Org 10,172 in Acute Stroke Treatment) criteria [19]. Expert neurologists assessed the clinical scales of the National Institutes of Health Stroke (NIHSS) upon admission and every 24 h during hospitalization.

For the first event, the neuroimaging study included computed tomography (CT), perfusion or angiography CT on admission, and CT or MRI at 48 h or at any time if neurological deterioration (≥4 points in the NIHSS) was detected. The presence of LA has been associated with small vessel disease, aging, and neurodegenerative conditions, and the severity of LA was assessed using the Fazekas scale, grading the severity changes in the white matter into grades (grade 0, no or minimal changes; grade 1, mild changes; grade 2, moderate changes; grade 3, severe changes) [20]. Expert neuroradiologists, blinded to clinical data, performed neuroimaging evaluations and classified LA.

### 2.4. Biomarker Determination

At the time of enrollment of the BICHUS biobank, venous blood samples were collected in vacutainer tubes (Becton Dickinson, San Jose, CA, USA) and centrifuged at 3000× *g* for 15 min at 4 °C. Serum was immediately aliquoted, frozen, and stored at −80 °C until analysis.

IL-6 and TNF-a were assessed as inflammation biomarkers, while sTWEAK was used as a marker of endothelial dysfunction. All biomarkers were evaluated in the first 24 h after the first stroke event. Using the IL-6 ELISA kit (Bender MedSystem, Wien, Austria), the minimum assay sensitivity was 1.6 pg/mL with an intra- and inter-assay coefficient of variation (CV) of 5.0% and 6.8%, respectively. TNF-a was measured using an immunodiagnostic IMMULITE 1000 System (Siemens Healthcare Global, Los Angeles, CA, USA). Minimum assay sensitivity was 1.7 pg/mL, with an inter-assay CV of 6.5% and an intra-assay CV of 3.5%. sTWEAK was assessed by experienced researchers in the Clinical Neurosciences Research Laboratory (blinded to clinical and radiological data) using a Human sTWEAK enzyme-linked immunosorbent assay (ELISA) kit (Elabscience, Houston, TX, USA). The minimum assay sensitivity was 4.69 pg/mL with an intra- and inter-assay CV of 5.06% and 5.21%, respectively. Serum NT-pro-BNP levels were measured by an electrochemiluminescence immunoassay (ELECSYS 2010 System; Roche Diagnostics GmbH, Mannheim, Germany). The rest of the laboratory markers were analyzed in the Biochemistry Laboratory of the University Clinical Hospital of Santiago de Compostela.

### 2.5. Statistical Analysis

This study reported categorical data as proportions and continuous data as mean and one standard deviation (SD) or median and interquartile range (IQR), according to the type of distribution determined by the Kolmogorov–Smirnov test for a sample with the correction of the significance of Lilliefors, as appropriate. The significance of the differences was estimated using Student’s t-test or the Mann–Whitney U test. A one-way analysis of variance (ANOVA) was used to compare differences between more than two groups. The qualitative variables were expressed as percentages, and for the differences, the chi-square test was used. Binary and multivariable logistic regression analyses were performed to assess associations. The results were expressed as an odds ratio (OR) with 95% confidence intervals (95% CIs). To detect the ability of biomarkers for stroke recurrence prediction, Receiver Operating Characteristic (ROC) curves were used, whereas the Youden index was used for choosing the cut-off points for biomarkers (the value with the maximum sensitivity and specificity). Significant values of *p* < 0.05 were considered. The analyses were performed with IBM SPSS v.25 for Mac.

## 3. Results

**Figure 2:**
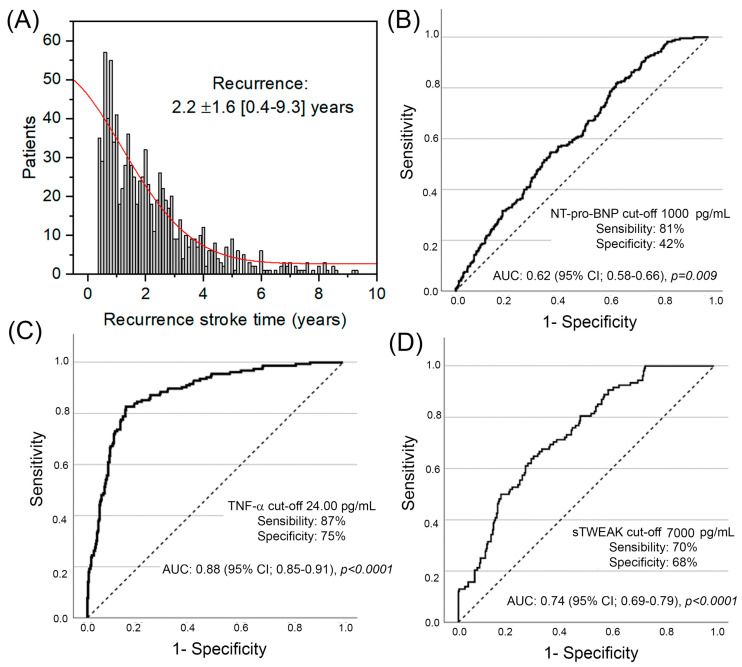
Time for stroke recurrence distribution until follow-up (red line), with a mean of 2.2 ± 1.6 years (**A**). The ROC curve for NT-pro-BNP showed a c-statistic of 0.62 (95% CI 0.58–0.66) for cardioembolic stroke prediction, with an optimal cut-off of >1000 pg/mL (**B**). The ROC curve for TNF-a showed a c-statistic of 0.88 (95% CI 0.85.0.91) for atherothrombotic stroke prediction, with an optimal cut-off of >24 pg/mL (**C**). The ROC curve for sTWEAK showed a c-statistic of 0.74 (95% CI 0.69–0.79) for intracerebral hemorrhage, with an optimal cut-off of >7000 pg/mL (**D**).

A total of 5038 patients were included in the final analysis with a mean follow-up time of 4.9 ± 3.3 years, a mean age of 72.3 ± 13.5 years, and 2791 (55.4%) males. The most frequent stroke subtypes were of cardioembolic origin at 29.8% (1502 patients), indeterminate origin at 25.1% (1264 patients), atherothrombotic origin at 18.6% (938 patients), ICH origin at 18.4% (928 patients), and lacunar strokes at 7.0% (354 patients). Stroke recurrences occurred in 18.4% of cases (927 patients). The mean time to recurrence was 2.2 (±1.6) years after the first stroke event (Figure 2A), being the most frequent subtype of recurrence: 43.9% (409 patients) for cardioembolic origin, 24.3% (225 patients) for ICH, 16.7% (156 patients) for atherothrombotic origin, 8% (74 patients) for indeterminate origin, and 6.5% (63 patients) for lacunar stroke. A significant deterioration was observed for the modified Rankin Scale score between the first and second stroke events (median 2 [IQR: 1–4] vs. median 3 [IQR: 1–5]; *p* < 0.001). Table 1 summarizes the descriptive analysis among patients who presented with/without stroke recurrences. Those patients with stroke recurrence presented a higher burden of traditional vascular risk factors and atrial fibrillation, a greater intake of previous antiplatelet (52.7% vs. 17.5%, *p* < 0.001) and anticoagulation (19.4% vs. 7.7%; *p* < 0.001) drugs, and a greater severity of NIHSS at admission (15 [9,10,11,12,13,14,15,16,17,18,19,20] vs. 13 [7,8,9,10,11,12,13,14,15,16,17,18]; *p* < 0.001), among others. Among potential molecular biomarkers for stroke recurrences, statistical significance was found for NT-pro-BNP (mean value 1426.8 ± 2353 vs. 1871.3 ± 2097; *p* < 0.001), IL-6 (mean value 14.55 ± 11.3 vs. 15.87 ± 11.8; *p* = 0.022), TNF-a (17.71 ± 7.5 vs. 18.98 ± 7.7; *p* = 0.002), and sTWEAK (5941.3 ± 3859 vs. 6708.1 ± 3939; *p* < 0.001). There were no differences in the presence of microalbuminuria between both groups. An ANOVA test was conducted to assess differences in biomarker levels across stroke subtypes, revealing statistically significant associations. NT-pro-BNP was significantly elevated in the cardioembolic subtype (*p* < 0.001), LA was associated with the lacunar subtype (*p* < 0.001), and TNF-α and IL-6 were linked to the atherothrombotic subtype (*p* < 0.001 and *p* < 0.01, respectively). Additionally, sTWEAK levels were significantly higher in intracerebral hemorrhage (ICH) cases (*p* < 0.001).

The first multivariable logistic regression model was performed for the selection of potential confounding variables (Table 2). It was observed that stroke recurrences were independently associated with the presence of arterial hypertension (adjusted OR 23.25; 95% CI: 1.71–315.34), ischemic heart disease (adjusted OR 13.58; 95% CI: 1.23–150.01), chronic antiplatelet therapy (adjusted OR 46.39; 95% CI: 6.30–341.38), and chronic anticoagulant therapy (adjusted OR 26.11; 95% CI:1.71–397.90).

### Multivariable Logistic Regression for Stroke Recurrence Etiology

Table 3 summarizes the findings of multivariable logistic regression models for stroke recurrence according to etiology. The presence of LA showed an independent association for lacunar stroke recurrence with an adjusted OR of 9.50 (95% CI: 3.12–28.93), whereas the presence of higher NT-pro-BNP levels after the first cardioembolic stroke showed a ROC curve of 0.62 (95% CI: 0.58–0.66) with an optimal cut-off of >1000 pg/mL (Figure 2B), which was independently associated with cardioembolic stroke recurrence (adjusted OR 1.80; 95% CI: 1.23–2.61). On the other hand, the persistence of high TNF-a levels after the second atherothrombotic stroke showed a ROC curve of 0.88 (95% CI: 0.85.0.91) with an optimal cut-off of >24 pg/mL (Figure 2C), which was independently associated with atherothrombotic stroke recurrence (adjusted OR 21.26; 95% CI: 12.42–37.59). Similarly, the persistence of high sTWEAK levels after the second ICH stroke showed a ROC curve of 0.74 (95% CI: 0.69–0.79) with an optimal cut-off of >7000 pg/mL (Figure 2D), which was independently associated with ICH stroke recurrence (adjusted OR 4.81; 95% CI: 2.86–8.07).

## 4. Discussion

Despite the best possible treatment after a stroke event and optimal management of stroke-associated vascular risk factors, recurrences remain high, with a direct impact burden on morbidity and mortality. Therefore, it is essential to consider the objective of increasing the effectiveness of secondary prevention as a priority [2,3,21].

Leukoaraiosis, a white matter lesion identified through neuroimaging, has gained recognition in recent decades as a crucial biomarker in the context of stroke [22]. It is clinically significant due to its association with an elevated risk of stroke incidence and recurrence, poor functional outcomes related to greater disability, and an increased risk of hemorrhagic transformation following IS [22,23]. LA is particularly prevalent in lacunar strokes and hemispheric intracerebral hemorrhages [24]. Although its pathogenesis remains poorly understood, it is considered a potential indicator of impaired collateral vessel health or reduced brain capacity for injury repair [22]. A recent systematic review encompassing 19 studies and 34,546 stroke patients reported that leukoaraiosis was linked with IS recurrence and, more notably, to ICH with a 30-fold increased risk. Furthermore, among patients with IS in the setting of AF, the presence of leukoaraiosis was associated with recurrence despite chronic anticoagulation therapy [25]. Furthermore, B-type natriuretic peptides are cardiac hormones associated with increasing volume/pressure overload, wall tension, or atrial dilatation in the setting of acute or chronic hemodynamic changes, being strongly related to prothrombotic arrhythmias, such as AF [26]. Previous research has found that NT-pro-BNP levels are independently associated with the cardioembolic stroke subtype and might be a useful biomarker for identifying cardioembolic origins in cryptogenic strokes [16,27,28]. In this setting, a meta-analysis that evaluated 23 studies with 2834 patients with IS found that NT-pro-BNP and BNP levels were significantly higher during the first 72 h after stroke onset. Thus, adding NT-pro-BNP to clinical models that included age, sex, and NIHH at admission, sensitivity, specificity, and predictive capability were improved to determine cardioembolic origin in patients with cardioembolic and cryptogenic stroke [29]. Moreover, different pro-inflammatory biomarkers such as C-reactive protein, TNF-a, or IL-6, among others, have been evaluated in the setting of stroke and appear to favor post-stroke complications, including stroke recurrences, poor clinical outcomes, and major adverse cardiovascular events, especially in the setting of large artery atherosclerosis, providing a potential therapeutic target for secondary stroke prevention [13,30,31,32]. In addition, sTWEAK belongs to the TNF superfamily, which is widely expressed in the neurovascular unit, leading to an upregulation after cerebral ischemia, favoring neuroinflammation and BBB disruption [33,34]. Thus, a maintained expression of sTWEAK might contribute to white matter disease associated with chronic cerebral ischemia, such as LA, and the increased risk for lacunar strokes [33,34,35], while previous research has found an independent association between sTWEAK levels and ICH, poor functional outcomes, and stroke recurrence after an IS event in patients who underwent reperfusion therapy [12,14].

This study aligned with the previous literature, showing that arterial hypertension and previous vascular disease are associated with an increased risk for overall stroke recurrences. Similarly, the use of chronic antiplatelet and anticoagulant therapy was associated with an increased risk of ICH, but with a protective role for cardioembolic stroke, as expected. In addition, although previous studies suggested a role for the presence of microalbuminuria in the prediction of hemorrhagic transformation after IS [36], the present study did not find any association between microalbuminuria and stroke recurrences. On the other hand, in this study, an independent association was observed between the presence of LA and lacunar stroke recurrence with a nearly 10-fold increased risk; however, in our cohort, there was no independent association for other stroke subtypes such as IHC. Elevated levels of NT-proBNP (>1000 pg/mL) were linked to an approximately 2-fold increased risk of cardioembolic stroke recurrence, which is in line with the previous literature, although predictive capability in our cohort was moderate (c-statistic of 0.62). Related to ICH, persistently elevated sTWEAK levels (>7000 pg/mL) after a hemorrhagic stroke were associated with a 5-fold increased risk of recurrent ICH, supporting the hypothesis of sTWEAK’s role and its implication in BBB disruption, promoting bleeding. Similarly, patients with a history of atherothrombotic stroke and persistently high TNF-a levels (>24 pg/mL) were observed to have a strong association (22-fold increased risk) with atherothrombotic stroke recurrence, highlighting the relevance of the inflammatory environment underlying atheromatous plaques. On the contrary, no significant association was observed between C-reactive protein levels and stroke recurrence.

The study findings suggest that monitoring specific biomarkers related to the etiology of stroke recurrence may be valuable in identifying patients at high risk of a recurrent event. Stroke recurrence remains a critical issue contributing to both mortality and morbidity, highlighting the urgent need for research aimed at optimizing secondary prevention measures.

This study presents some limitations. First, it is a retrospective analysis based on a prospective registry and single-center study without pre-clinical data to support the molecular mechanism involved between biomarkers and outcome. Second, the molecular biomarkers analyzed were measured from frozen samples at different times and by various researchers, which could introduce variability due to the potential degradation of biomolecules affecting the viability of subsequent analysis, even under optimal storage conditions. Third, the inter-rater reliabilities were not assessed regarding neuroimaging evaluation for the presence and grade of LA. Fourth, there were no specific variables to assess potential concomitant causes of inflammation. However, the study also demonstrates notable strengths. Firstly, although retrospective, potential biases were minimized by including all consecutive patients registered at our center for their first event. For recurrent cases, data were comprehensively gathered from the entire public regional network (the private network in our region is relatively small), reflecting real-world clinical data. Secondly, molecular biomarker data were analyzed independently, with researchers blinded to clinical and neuroradiological information. Thirdly, long-term stroke recurrences (a mean of nearly 5 years of follow-up) were evaluated in terms of real-world data. Lastly, the study offers a plausible pathophysiological rationale connecting molecular biomarkers with an increased risk of stroke recurrence.

## 5. Conclusions

Molecular and neuroimaging biomarkers may help predict specific stroke recurrence etiologies. In our cohort, leukoaraiosis was a predictor of lacunar stroke recurrence, increasing the risk 10-fold. Elevated NT-pro-BNP levels (above 1000 pg/mL) were associated with nearly a 2-fold increased risk of cardioembolic stroke recurrence. Additionally, persistently high levels of TNF-α (above 24 pg/mL) and sTWEAK (above 7000 pg/mL) were linked to a 2-fold and 5-fold increased risk of atherothrombotic and intracerebral hemorrhage (ICH) stroke recurrences, respectively. Further studies with a robust prospective design are needed to confirm these findings.

## Figures and Tables

**Table 1:** Descriptive analysis among patients who presented with/without stroke recurrences.

	No Stroke Recurrences n = 4111	Stroke Recurrences n = 927	*p*
Age, years	71.4 ± 14	76.1 ± 11	<0.001
Women, %	44.6	44.4	0.943
Hypertension, %	61.6	71.3	<0.001
Dyslipidemia, %	33.6	45.0	<0.001
Diabetes, %	22.4	29.3	<0.001
Smokers, %	16.0	12.2	0.002
Atrial fibrillation, %	19.1	30.0	<0.001
Ischemic heart disease, %	10.3	13.9	0.002
Heart failure, %	4.0	5.8	0.013
Antiplatelet (chronic), %	17.5	52.7	<0.001
Anticoagulant (chronic), %	7.7	19.4	<0.001
Glycemia *, mg/dL	136.9 ± 55	140.8 ± 63	0.077
Leukocytes *, ×10^3^/mL	9.0 ± 3.2	9.7 ± 3.3	<0.001
C-reactive protein *, mg/dL	3.9 ± 4.4	4.6 ± 4.5	<0.001
Glycosylated hemoglobin, %	6.1 ± 2.2	6.1 ± 1.6	0.354
LDL cholesterol, mg/dL	114.4 ± 43.5	98.2 ± 36.6	<0.001
HDL cholesterol, mg/dL	41.5 ± 19.1	39.7 ± 14.6	0.035
Triglycerides, mg/dL	120.2 ± 64.2	107.6 ± 55.2	<0.001
IMT, mm	0.9 ± 0.7	1.1 ± 1.1	0.062
Leukoaraiosis, %	41.8	57.4	<0.001
Degree of leukoaraiosis, %			<0.001
No	58.6	42.6	
Fazecas I	40.6	50.8	
Fazecas II	0.6	4.8	
Fazecas III	0.1	1.7	
NIHSS at admission	13 [7, 18]	15 [9, 20]	<0.001
Etiology of stroke			<0.001
PIC, %	17.7	21.6	
Cardioembolic, %	28.6	35.2	
Atherothrombotic, %	18.9	17.4	
Lacunar, %	7.7	4.0	
Indeterminate, %	26.0	21.1	
Others, %	1.1	0.8	
Any reperfusion therapy, %			0.067
Systemic fibrinolysis, %	22.6	26.3	
Thrombectomy, %	5.5	3.7	
Combination therapy, %	0.5	1.2	
NT-pro-BNP *, pg/mL	1426.8 ± 2353.3	1871.3 ± 2097.1	<0.001
TNF-a *, pg/mL	17.71 ± 7.5	18.98 ± 7.7	0.002
IL-6 *, pg/mL	14.55 ± 11.3	15.87 ± 11.8	0.022
IL-10 *, pg/mL	6.79 ± 4.7	7.07 ± 4.5	0.277
sTWEAK *, pg/mL	5941.3 ± 3859.5	6708.1 ± 3939.1	<0.001
Microalbuminuria *, mg/g	7.3 ± 26.7	8.1 ± 26.8	0.555

* In the first 24 h from admission. IL: interleukin; IMT: intima-media thickness; NIHSS: National Institutes of Health Stroke Scale; sTWEAK: soluble tumor necrosis factor-like weak inducer of apoptosis; TNF-a: tumoral necrosis factor-alpha.

**Table 2:** Binary and multivariable logistic regression analysis for comorbidities and stroke recurrence (dependent variable).

	Binary			Multivariable		
	OR	CI 95%	*p*	aOR *	CI 95%	*p*
Age	1.03	1.02–1.03	<0.001	0.98	0.91–1.07	0.752
Hypertension	1.54	1.33–1.80	<0.001	23.25	1.71–315-34	0.018
Diabetes	1.44	1.23–1.68	<0.001	1.19	0.25–5.72	0.829
Smoker	0.71	0.57–0.88	0.002	0.26	0.05–1.49	0.132
Dyslipidemia	1.61	1.40–1.86	<0.001	2.14	0.52–8.85	0.295
IHD	1.41	1.15–1.73	0.001	13.58	1.23–150.01	0.033
Previous AF	1.81	1.55–2.11	<0.001	3.87	0.34–44.59	0.278
Heart failure	1.49	1.09–2.03	0.012	1.49	0.14–16.23	0.739
Antiplatelets	5.25	4.53–6.09	<0.001	46.39	6.30–341.38	<0.001
Anticoagulants	2.89	2.39–3.51	<0.001	26.11	1.71–397.90	0.019
NIHSS ^†^	1.03	1.03–1.04	<0.001	1.04	0.94–1.15	0.442
LDL cholesterol	0.99	0.98–0.099	<0.001	1.01	0.99–1.03	0.176
HDL cholesterol	0.99	0.98–0.99	0.032	1.00	0.96–1.05	0.840
Triglycerides	0.99	0.99–0.99	<0.001	1.00	0.99–1.02	0.337
Leucocytes ^†^	1.06	1.04–1.08	<0.001	1.19	0.98–1.46	0.076
CRP ^†^	1.03	1.01–1.05	<0.001	0.90	0.76–1.08	0.261
IL-6 ^†^	1.03	1.01–1.01	<0.001	1.02	0.95–1.09	0.572

* Adjusted odds ratio. ^†^ Measured in the first 24 h from admission. AF: atrial fibrillation; IHD: ischemic heart disease; CRP: C-reactive protein; IL: interleukin; NIHSS: National Institutes of Health Stroke Scale.

**Table 3:** Multivariable logistic regression analysis for stroke recurrences according to etiology.

Multivariable			
	aOR *	CI 95%	*p*
Lacunar stroke			
Hypertension	4.98	2.04–12.25	<0.001
Ischemic heart disease	1.44	0.42–4.98	0.565
Antiplatelets	1.19	0.53–2.67	0.681
Anticoagulation	0.17	0.02–1.36	0.095
Leukoaraiosis	9.50	3.12–28.93	<0.001
Cardioembolic stroke			
Hypertension	1.06	0.75–1.49	0.759
IHD	1.51	0.95–2.41	0.083
Antiplatelets	0.25	0.17–0.35	<0.001
Anticoagulation	0.19	0.13–0.29	<0.001
NT-pro-BNP ^†^ >1000 pg/mL	1.80	1.23–2.61	0.002
Atherothrombotic stroke			
Hypertension	1.49	0.89–2.49	0.129
IHD	1.29	0.72–2.33	0.390
Antiplatelets	1.89	1.14–3.15	0.014
Anticoagulation	0.79	0.41–1.55	0.500
TNF-a ^†^ >24 pg/mL	21.61	12.42–37.59	<0.001
Intracerebral hemorrage			
Hypertension	1.33	0.76–2.32	0.316
IHD	2.16	0.85–5.45	0.104
Antiplatelets	2.04	1.16–3.57	0.013
Anticoagulation	2.27	1.11–4.65	0.002
sTWEAK ^†^ >7000 pg/mL	4.81	2.86–8.07	<0.001

* Adjusted odds ratio. ^†^ Measured in the first 24 h from admission. IHD: ischemic heart disease; TNF-a: tumor necrosis factor-alpha; sTWEAK: soluble tumor necrosis factor-like weak inducer of apoptosis.

## Data Availability

The statistical analysis plan is available upon request. The data bank is not available for legal and ethical reasons.
